# Rapid-Deployment Versus Sutured Aortic Valve Replacement Through Right Anterior Thoracotomy: A Single-Team Retrospective Comparison of Operative Efficiency in 192 Consecutive Patients

**DOI:** 10.3390/life16091573

**Published:** 2026-09-21

**Authors:** Łukasz Jaworski, Krzysztof Jarmoszewicz, Adam R. Kowalówka, Marian Burysz, Sebastian Batkiewicz, Krzysztof Roszak

**Affiliations:** 1 Department of Cardiac Surgery, F. Ceynowa Specialist Hospital, 84-200 Wejherowo, Polandkrzych.roszak@gmail.com (K.R.); 2 Department of Cardiac Surgery, Upper-Silesian Heart Center, Medical University of Silesia, 40-635 Katowice, Poland; 3 Department of Cardiac Surgery, Regional Specialist Hospital, 86-300 Grudziadz, Poland; marianburysz12@gmail.com

**Keywords:** aortic valve replacement, rapid-deployment valve, right anterior thoracotomy, minimally invasive cardiac surgery, cross-clamp time, Edwards Intuity

## Abstract

Minimally invasive aortic valve replacement through a right anterior thoracotomy is limited by prolonged operative times. We assessed whether a rapid-deployment prosthesis reduces those times and improves early outcomes when surgical access, team and era are held constant. Single-centre retrospective cohort study of 204 consecutive isolated aortic valve replacements performed through a right anterior thoracotomy by one surgical team between 2023 and 2025. Patients receiving a mechanical prosthesis (*n* = 5) or a 29 mm sutured valve (*n* = 7, a size not manufactured for the study device) were excluded, leaving 192 patients: 70 received the Edwards Intuity rapid-deployment valve and 122 a conventional sutured bioprosthesis. Prosthesis choice was at the surgeon’s discretion. Primary endpoints were aortic cross-clamp and cardiopulmonary bypass time. Groups were compared by multivariable regression adjusted for age, sex, EuroSCORE II, NYHA class, ejection fraction and implanted valve size, with robust standard errors for continuous outcomes and Firth-penalised logistic regression for binary outcomes. Aortic cross-clamp time was shorter with the Intuity (median 61.0 [54.0–75.0] versus 91.0 [76.0–108.5] min; adjusted difference −26.9 min, 95% CI −33.7 to −20.1; *p* < 0.001), as was cardiopulmonary bypass time (103.5 [84.2–115.0] versus 131.0 [115.0–153.0] min; adjusted difference −29.4 min, −38.7 to −20.2; *p* < 0.001). Mean and peak transvalvular gradients were lower (adjusted differences −5.52 mmHg, −6.80 to −4.25, and −11.50 mmHg, −13.90 to −9.10; both *p* < 0.001) and hospital stay was shorter by 1.4 days (−2.4 to −0.4; *p* = 0.009). In-hospital mortality was 1/70 versus 1/122 (*p* = 1.000). Permanent pacemaker implantation occurred in 6/70 (8.6%) versus 2/122 (1.6%) (unadjusted *p* = 0.053) and paravalvular leak of any grade in 7/69 (10.1%) versus 8/121 (6.6%); with eight and fifteen events respectively, neither endpoint could be estimated with useful precision; these non-significant comparisons should not be read as evidence of comparable safety. Within a single access, team and era, the rapid-deployment prosthesis was associated with substantially shorter operative times, lower gradients and shorter hospital stay, with similar observed in-hospital mortality (1/70 versus 1/122); with only two deaths overall this comparison cannot establish equivalence. These findings describe operative efficiency rather than demonstrated clinical superiority, and the safety comparisons remain underpowered.

## 1. Introduction

Aortic stenosis is the most prevalent valvular lesion in older adults, and aortic valve replacement is the guideline-recommended treatment for severe symptomatic disease [1,2,3,4]. Transcatheter replacement has been extended to intermediate- and low-risk patients [5,6], and the current European guidelines now favour transfemoral transcatheter implantation from the age of 70 years, while surgical replacement remains the standard in patients below that age at low surgical risk and in those with bicuspid anatomy, supported by its durable long-term outcomes [2,7]. Among surgical options, minimally invasive access has been associated with reduced transfusion, shorter ventilation, and shorter hospital stay relative to full sternotomy, with comparable mortality [8,9]. The right anterior thoracotomy appears to confer the largest of these benefits: several series report lower postoperative atrial fibrillation and shorter hospital stay than both sternotomy and mini-sternotomy [10,11]. The technique is supported by expert consensus, defined by computed-tomography selection criteria [12] and an initial forty-five to fifty case learning period [13].

This access nonetheless carries one recurring limitation. The benefits of the right anterior thoracotomy are accompanied by longer operative times. Propensity-matched comparisons with full sternotomy consistently report prolonged cross-clamp and cardiopulmonary bypass times for the minimally invasive approach [9,14]. This finding is relevant because longer ischaemic and bypass times have been associated with increased morbidity and mortality after aortic valve replacement [15,16]. The extent of the penalty also varies with anatomy: aortic dextroposition and a small aortic annulus are each associated with longer cross-clamp times in the right anterior approach [17], and assessment of the aortic root and ascending aorta should follow current guideline standards for the aortic organ [3]. The operative-time cost of minimally invasive access therefore offsets part of its recovery benefit. Shortening this time without compromising the approach is a key technical objective.

The rapid-deployment valve was engineered to remove this penalty. The Edwards Intuity pairs a Carpentier-Edwards Perimount pericardial valve with a balloon-expandable subannular frame, so that three guiding sutures replace a full ring of annular sutures [18,19]. This design shortens aortic cross-clamp time by roughly thirty minutes relative to conventional sutured valves [20]. The reduction is largest precisely where access is most constrained: through the right anterior thoracotomy, rapid-deployment valves cut cross-clamp and bypass times by a third or more [21,22,23]. The saving carries no haemodynamic cost, as the Intuity achieves low transvalvular gradients and large effective orifice areas across pivotal trials [24,25]. Combining the valve with the approach should therefore neutralise the operative-time objection to minimally invasive surgery, a rationale consistent with broader reviews of patient selection for rapid-deployment and sutureless valves across surgical contexts [26]. Yet only a handful of dedicated right anterior thoracotomy series have tested the combination, and the case for the valve rests on more than time alone [21,27,28].

The valve’s time advantage is accompanied by an increased risk in conduction disturbance. Because the subannular frame lies close to the membranous septum, the Intuity series have reported permanent pacemaker rates of eight to fourteen percent, compared with one to five percent for conventional sutured valves [29,30,31]. This carries clinical weight, as pacemaker implantation after surgical aortic valve replacement has been independently associated with reduced long-term survival and increased heart-failure hospitalisation [32,33]. Consequently, the operative-time benefit may not consistently translate into improved outcomes: in straightforward isolated replacement, rapid-deployment valves shortened cross-clamp time by nearly thirty minutes but showed no survival advantage and a markedly higher pacemaker rate [34]. Registry data describe a similar pattern [35,36]. Whether the valve’s benefits outweigh this risk may depend on context, including the type of surgical access.

Sutureless valves have been compared against sutured prostheses within the right anterior thoracotomy for the self-expanding, sutureless Perceval [22], and for the balloon-expandable Intuity itself, in a single-centre series of sixty-eight patients [21]—to date the largest dedicated comparison of these two prosthesis types within this access. Larger comparisons of the Intuity against sutured valves exist but pool surgical accesses, leaving the valve’s effect confounded with the approach [35,37,38], and the largest multicentre registry to date pools sutureless with rapid-deployment valves as well as surgical accesses, confounding both the prosthesis and the approach at once [39]. In this study, we therefore compared the Edwards Intuity with conventional sutured bioprostheses in 192 patients, drawn from 204 consecutive aortic valve replacements performed through the right anterior thoracotomy by a single team during a concurrent period, adjusting for baseline differences between groups. Because access, team and era are common to both groups, this design isolates the prosthesis as the principal variable distinguishing them more closely than pooled multicentre comparisons, but it primarily evaluates operative efficiency and early safety rather than a clinically unresolved efficacy question; whether the valve’s association with clinical secondary outcomes is mediated by the shorter operative time itself or reflects an independent device-specific effect is addressed as an open question in the discussion.

## 2. Materials and Methods

### 2.1. Study Design and Population

This was a single-centre, retrospective cohort study conducted at the Department of Cardiac Surgery, Wejherowo, Poland. We reviewed consecutive patients who underwent isolated surgical aortic valve replacement through a right anterior thoracotomy between 2023 and 2025. Over this concurrent period the same surgical team implanted either a rapid-deployment valve or a conventional sutured bioprosthesis, with valve choice at the surgeon’s discretion rather than a fixed anatomical or clinical algorithm; in practice this discretion was informed predominantly by device availability and surgeon preference at the time of operation, together with anatomical suitability (annulus size within the Intuity’s manufactured range), rather than by a prospectively defined selection protocol, so the potential for unmeasured selection bias in prosthesis choice cannot be excluded.

Patients were assigned to two groups by implanted prosthesis: Intuity (rapid-deployment) and Suture (conventional sutured bioprosthesis). Within the Suture group, the implanted valve was the Medtronic Hancock II in 95 patients (77.9%), a Carpentier-Edwards Perimount or Perimount Magna/Magna Ease in 11 (9.0%), the Medtronic Avalus in 9 (7.4%), a St Jude/Abbott Epic in 4 (3.3%), the Edwards Resilia in 2 (1.6%), and the Medtronic Mosaic in 1 (0.8%). Because the study compares two bioprostheses, patients receiving a mechanical valve were excluded (*n* = 5). Patients receiving a 29 mm sutured prosthesis were also excluded (*n* = 7), as the Intuity is not manufactured in this size and such patients therefore have no possible matched comparator. Of 204 patients assessed (70 Intuity and 134 non-Intuity, of whom 129 received a sutured bioprosthesis and 5 a mechanical prosthesis), 192 remained eligible after these exclusions (70 Intuity, 122 Suture). All exclusions were recorded a priori (Table S1).

### 2.2. Surgical Technique

All procedures were performed through a right anterior thoracotomy in the second or third intercostal space, with direct vision and without video assistance throughout. The intercostal space was selected individually from the preoperative computed-tomography scan: the space vertically aligned with the mid-point of the aortic valve/proximal ascending aorta, avoiding overlying rib or costal cartilage calcification and excessive angulation to the annulus, was chosen, which was the third space in patients with a more caudally positioned aortic root and the second space in patients with a more cephalad root; the final confirmation was made by intraoperative trans-illumination and echocardiographic assessment of alignment with the aortic annulus. All procedures in this series were performed under direct vision without video or endoscopic assistance; the skin incision did not exceed 6 cm, and exposure was maintained with a soft-tissue retractor of 4–6 cm blade length together with a small rib spreader, neither of which is required for the narrower, endoscopically assisted wound (typically 2–4 cm) used in video-assisted variants of this access elsewhere; this distinction is noted because it is a direct-vision, not an endoscopic, minimally invasive technique. Preoperative computed tomography was used to assess aortic root geometry, chest anatomy, and femoral vessel calibre for access planning; computed-tomography angiography of the iliofemoral vessels was obtained in all patients before peripheral cannulation. Cardiopulmonary bypass was established by peripheral femoral arterial and venous cannulation in all patients. Cannulation was performed percutaneously by the Seldinger technique, with vascular closure achieved using the Proglide 2× technique in standard cases. In the presence of atherosclerotic plaque, whether hard or soft, in the iliofemoral axis, femoral arterial cannulation was avoided and surgical cannulation of the subclavian artery was performed instead. Transoesophageal echocardiography was used routinely to confirm the guidewire position during both arterial and venous cannulation. The ascending aorta was cross-clamped transthoracically. Myocardial protection was achieved with antegrade crystalloid cardioplegia in 121 patients (95 Suture, 26 Intuity) and antegrade blood cardioplegia in 70 patients (26 Suture, 44 Intuity), with cardioplegia type not recorded in 1 patient. Cardioplegia type was not a matching covariate, and its distribution differed markedly between groups.

After a standard transverse or oblique aortotomy, the native valve was excised and the annulus debrided and sized in both groups. Intuity valves were implanted with three guiding sutures placed at the commissures, the valve positioned within the annulus, and the polyester sealing skirt expanded by transient balloon inflation of the subannular frame under a fixed pressure and duration per the manufacturer’s instructions, after which the guiding sutures were tied. Sutured bioprostheses were implanted with single sutures placed circumferentially around the annulus and tied sequentially. Valve sutures were fastened routinely with the Cor-Knot automated device (LSI Solutions, Victor, NY, USA) in the Suture group; because this device itself shortens suturing time, the comparator arm represents an accelerated sutured technique rather than conventional hand-tied implantation, and the magnitude and generalisability of the operative-time difference reported below should be interpreted against this already-accelerated comparator rather than against hand-tied suturing. The aortotomy was closed in both groups with a running suture, and de-airing and weaning from bypass followed standard practice. Intraoperative transoesophageal echocardiography was used routinely to screen for paravalvular leak and valve function at the end of the procedure.

### 2.3. Endpoints

The primary endpoints were aortic cross-clamp time and cardiopulmonary bypass time, the operative-efficiency measures most directly related to rapid-deployment; both are process measures rather than clinical outcomes and are interpreted as such throughout. Secondary endpoints, each analysed with covariate adjustment, were in-hospital mortality, permanent pacemaker implantation, new-onset atrial fibrillation, paravalvular leak of any grade, postoperative mean and peak transvalvular gradients, and length of hospital stay. Paravalvular leak severity (trace/mild/moderate/severe) is reported descriptively as trace or mild in all affected patients in both groups; no patient in either group had moderate or severe paravalvular leak. Because the primary any-grade comparison above is numerically dominated by trace leak, which is of limited clinical significance, this severity breakdown is reported descriptively rather than as a formally adjusted secondary endpoint, given the very small per-category counts. A third group comprised additional perioperative complications: re-exploration for chest-wound bleeding, perioperative blood transfusion, transient ischaemic attack or stroke, pneumonia or pulmonary infection, pleural effusion requiring drainage, and pleural haematoma. As their counts are sparse, they are compared between groups without adjustment using Fisher’s exact test.

### 2.4. Statistical Analysis

Baseline characteristics are reported as mean ± standard deviation for continuous variables and counts (percentages) for categorical variables, compared with the *t*-test and chi-squared test respectively. EuroSCORE II is strongly right-skewed and is given as the median [interquartile range] with the Wilcoxon rank-sum test. Ejection fraction is summarised as a mean rather than a median because two-thirds of patients have a value of exactly 60%, which collapses the interquartile range into a single point; it is additionally dichotomised at 50%. NYHA class and implanted valve size are treated as categorical throughout; all observed levels are shown, and because several contain no Suture patient, these two variables, along with the near-constant aortic stenosis indicator, are compared with Fisher’s exact test.

Groups were compared for primary and secondary outcomes by multivariable regression adjusting for six confounders of prosthesis choice: age, male sex, EuroSCORE II, NYHA class, ejection fraction, and implanted valve size. These covariates were specified before analysis on clinical grounds as the factors plausibly influencing prosthesis selection and were not selected on the basis of their baseline *p*-values. Aortic stenosis was not adjusted for: it was present in approximately 99% of both groups. Covariates were entered additively. NYHA class was dichotomised as class III versus class I or II, and valve size entered as four categories (≤21, 23, 25 and 27 mm); in each case the sparse extreme category was collapsed so that every level contained patients from both groups.

Continuous endpoints were analysed by linear regression with robust (heteroscedasticity-consistent) standard errors. Cross-clamp time, cardiopulmonary bypass time and length of stay had markedly right-skewed residuals (skewness +1.4, +1.1 and +5.9) and were log-transformed; transvalvular gradients showed only moderate residual skewness that log-transformation over-corrected and were left untransformed. The group effect is reported as an adjusted difference on the original scale and as the ratio of geometric means estimated directly by the log-scale model, obtained with the emmeans package by contrasting each group’s marginal mean averaged over the observed covariate distribution; intervals for back-transformed differences were calculated using the Delta method with the robust variance carried through.

Binary endpoints were analysed by Firth-penalised logistic regression, with group effects reported as adjusted odds ratios with profile-penalised confidence intervals, chosen because event counts were low relative to the nine predictor terms in the model. For permanent pacemaker implantation, however, eight events across the whole cohort fall far below any accepted events-per-variable threshold, so the unadjusted comparison is reported as the primary estimate for this endpoint and the penalised adjusted odds ratio is given only for completeness. In-hospital mortality was not modelled: with only two deaths it is reported as unadjusted counts compared with Fisher’s exact test.

Analyses were complete-case. Missing data affected operative times (2 patients), paravalvular leak and length of stay (2 patients each) and echocardiographic gradients (4 patients); these were isolated omissions in the operative and echocardiographic records, unrelated to outcome, and no imputation was performed. One sensitivity analysis was specified: every model was re-fitted with the seven patients who received a 29 mm sutured prosthesis retained (*n* = 199). Because the study analysed all consecutively eligible patients rather than a prospectively sized sample, no a priori power calculation was performed. As a post hoc indication of precision, the observed event counts for the two safety endpoints’ nearest significance permit reliable detection only of large effects: with 8 pacemaker events across 192 patients and 15 paravalvular leak events across 190, the 95% confidence intervals for the adjusted odds ratios (0.67–28.17 and 0.81–10.85, respectively) span more than a ten-fold and thirteen-fold range, so a true doubling or trebling of either risk cannot be excluded by this dataset. No formal correction for multiple comparisons was applied; inference was instead structured hierarchically, with cross-clamp and bypass time as the pre-specified primary endpoints and all remaining endpoints secondary and hypothesis-generating. Because prosthesis use was not randomly allocated over the three-year study period, calendar time and case sequence are potential confounders of the primary operative-time endpoints in particular, since the covariate-adjustment framework above does not include calendar time or cumulative case number as a modelled covariate. A two-sided *p*-value below 0.05 was considered significant. Analyses used R version 4.3.3 (R Foundation for Statistical Computing, Vienna, Austria) with the tableone (0.13.2), sandwich (3.1.1), lmtest (0.9.40), logistf (1.26.1) and emmeans (1.11.0) packages.

## 3. Results

Of 204 consecutive patients assessed, 70 received the Edwards Intuity valve and 134 received another prosthesis, of whom 129 received a conventional sutured bioprosthesis and five a mechanical valve. Twelve patients were excluded: five had received a mechanical prosthesis, and seven a 29 mm sutured valve. This left 192 patients, 70 in the Intuity group and 122 in the Suture group. The groups differed at baseline in the EuroSCORE II (*p* = 0.032) and NYHA class (*p* = 0.004), and cardioplegia type differed markedly (crystalloid in 77.9% of Suture versus 37.1% of Intuity patients, *p* < 0.001). Age, sex, and ejection fraction did not differ, and 37 patients (19.3%) had an ejection fraction of 50% or less. Implanted valve size differed in distribution without reaching significance (*p* = 0.081), the Suture group being concentrated at 23 mm and the Intuity group at 25 mm (Table 1). All subsequent comparisons are adjusted for age, sex, EuroSCORE II, NYHA class, ejection fraction, and valve size.

### 3.1. Operative Times

**Figure 1:**
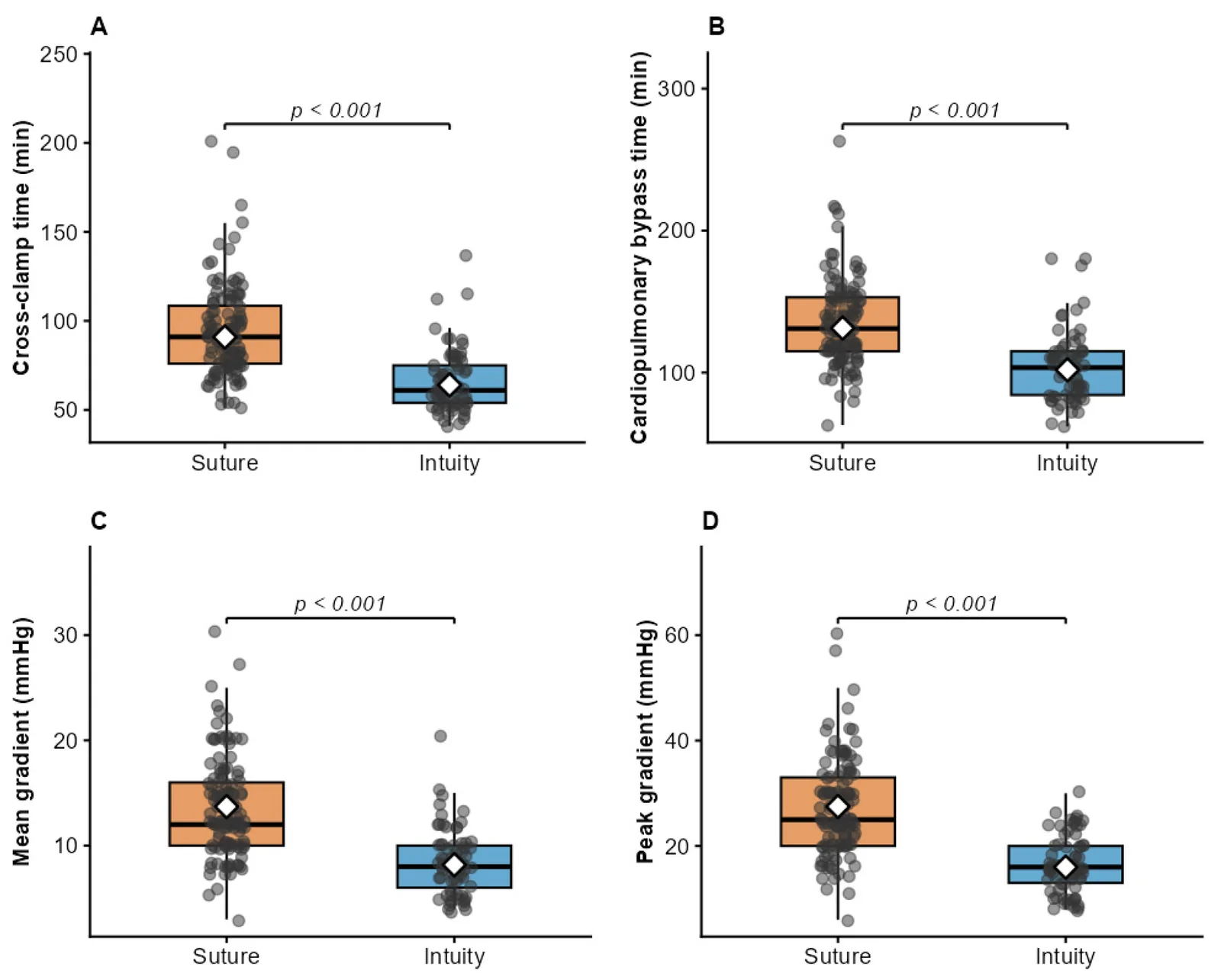
Operative times and postoperative transvalvular gradients. Box plots (median, interquartile range, and 1.5 × IQR whiskers) with individual patient values overlaid as jittered points. (**A**) Aortic cross-clamp time and (**B**) cardiopulmonary bypass time, both *n* = 190. (**C**) Mean and (**D**) peak postoperative transvalvular gradient, both *n* = 188. White diamonds mark the covariate-adjusted mean for each group—a geometric mean in (**A**,**B**), whose outcomes are log-transformed, and an arithmetic mean in (**C**,**D**), which are not. Brackets indicate the adjusted *p*-value for the group effect.

**Figure 2:**
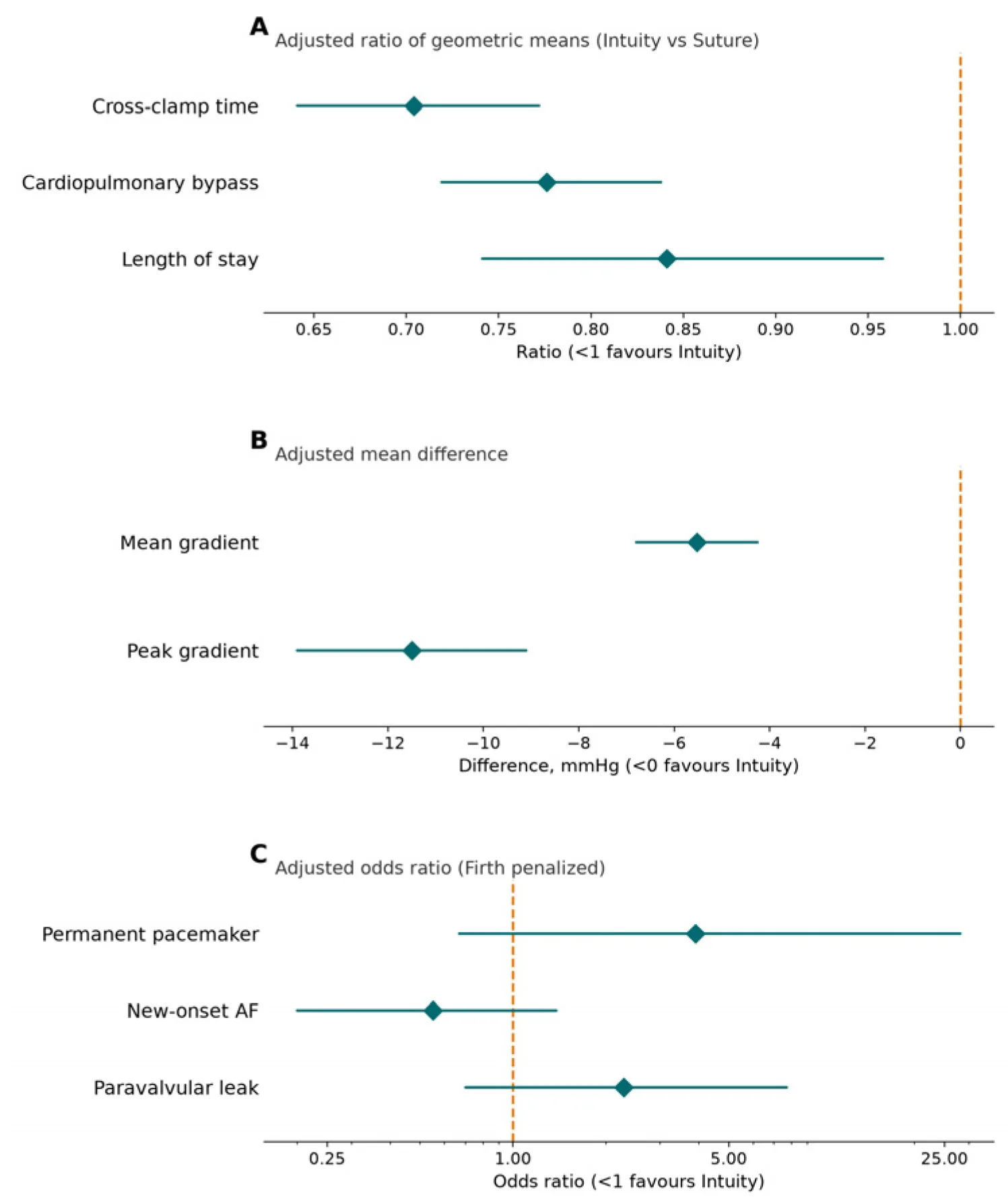
Adjusted group effects across all endpoints, from the models reported in Table 2, Table 3 and Table 4 (all adjusted for age, sex, EuroSCORE II, NYHA class, ejection fraction, and valve size). Analysed numbers differ by endpoint: *n* = 192 for the binary in-hospital outcomes, *n* = 190 for operative times and length of stay, and *n* = 188 for the gradients. Dashed lines mark the null. (**A**) Operative times and length of stay as adjusted ratios of geometric means from log-transformed linear models. (**B**) Transvalvular gradients as adjusted mean differences in mmHg from untransformed linear models. (**C**) In-hospital binary outcomes as adjusted odds ratios from Firth-penalised logistic regression. Paravalvular leak is included in panel (**C**) using the re-fitted adjusted odds ratio (OR 2.28, 95% CI 0.70–7.72) calculated on the corrected Suture-group any-grade count (8/121). Panels (**A**,**C**) use logarithmic axes on which the null is 1, whereas (**B**) is linear with a null of 0. In-hospital mortality is not shown: with two events no adjusted estimate is supportable.

Aortic cross-clamp time was shorter in the Intuity group, at a median of 61.0 min [54.0–75.0] versus 91.0 [76.0–108.5]; the adjusted difference was −26.9 min (95% CI −33.7 to −20.1), an adjusted ratio of geometric means of 0.70 (0.64–0.77; *p* < 0.001). Cardiopulmonary bypass time was likewise shorter, at 103.5 min [84.2–115.0] versus 131.0 [115.0–153.0], an adjusted difference of −29.4 min (−38.7 to −20.2) and a ratio of 0.78 (0.72–0.84; *p* < 0.001) (Table 2, Figure 1A,B and Figure 2).

Prosthesis use was not evenly distributed across the three-year study period: Intuity implantation became more frequent in the later part of the cohort as the team’s cumulative experience with this access grew, consistent with the recognised forty-five-to-fifty case learning curve for right anterior thoracotomy [13]. Because operative times for both prostheses would be expected to fall with accumulating case experience independent of device, calendar time and case sequence are plausible confounders of the cross-clamp and bypass-time comparison reported above. A calendar-period-stratified or case-sequence-adjusted sensitivity analysis was not part of the pre-specified statistical plan and could not be added retrospectively within the scope of this analysis; we therefore present the operative-time findings with this important caveat and treat the possibility of learning-curve confounding as a central limitation of the study, rather than concluding that the prosthesis itself is solely responsible for the observed time saving.

### 3.2. In-Hospital Secondary Outcomes

In-hospital mortality was one of 70 (1.4%) in the Intuity group and one of 122 (0.8%) in the Suture group (*p* = 1.000); with only two deaths in the whole cohort, this comparison is reported descriptively as observed event rates rather than as evidence of comparable mortality risk, and no adjusted or between-group statistical inference is drawn from it. Length of hospital stay was shorter in the Intuity group, at 6.0 days [6.0–7.0] versus 8.0 [7.0–10.0]—an adjusted difference of −1.4 days (−2.4 to −0.4) and a ratio of 0.84 (0.74–0.96; *p* = 0.009) (Table 3). None of the remaining in-hospital outcomes differed significantly after adjustment. Permanent pacemaker implantation was more frequent in the Intuity group (6/70, 8.6%, versus 2/122, 1.6%; unadjusted *p* = 0.053); with eight events in the whole cohort this endpoint cannot be estimated with useful precision, and the penalised adjusted odds ratio of 3.90 (95% CI 0.67–28.17, *p* = 0.134) is reported for completeness only. New-onset atrial fibrillation was less frequent in the Intuity group (7/70, 10.0%, versus 28/122, 23.0%), significant before adjustment (*p* = 0.032) but not after it (adjusted odds ratio 0.55, 0.20–1.38, *p* = 0.208). Paravalvular leak of any grade was more frequent in the Intuity group before adjustment (7/69, 10.1%, versus 8/121, 6.6%; unadjusted *p* = 0.411) using Fisher’s exact test; the Suture-group count was corrected after an echocardiographic re-review from 6/121 to 8/121, and the multivariable Firth model was re-fitted on the corrected count using the full patient-level dataset, yielding an adjusted odds ratio of 2.28 (95% CI 0.70–7.72, *p* ‡ = 0.168), consistent in direction and magnitude with the pre-correction estimate and likewise not statistically significant), consistent with no significant unadjusted difference; all affected patients in both groups had trace or mild leak, with none moderate or severe (trace: three Intuity versus six Suture; mild: four Intuity versus two Suture; Table 4). 

**Table 3:** In-hospital secondary outcomes.

Outcome	Intuity (*n* = 70)	Suture (*n* = 122)	Adjusted Effect (95% CI)	*p*	*p* ‡
In-hospital mortality	1/70 (1.4)	1/122 (0.8)	Not modelled (2 events)	1.000	—
Permanent pacemaker	6/70 (8.6)	2/122 (1.6)	OR 3.90 (0.67–28.17)	0.053	0.134
New-onset atrial fibrillation	7/70 (10.0)	28/122 (23.0)	OR 0.55 (0.20–1.38)	0.032	0.208
Paravalvular leak (any)	7/69 (10.1)	8/121 (6.6)	OR 2.28 (0.70–7.72)	0.411	0.168
Length of stay (days)	6.0 [6.0, 7.0]	8.0 [7.0, 10.0]	−1.4 (−2.4 to −0.4)	<0.001	0.009

Values are *n* (%) for binary outcomes and median [interquartile range] for length of stay. The unadjusted *p* is from Fisher’s exact test for binary outcomes and the Wilcoxon rank-sum test for length of stay. Adjusted effects are odds ratios from Firth-penalised logistic regression with profile-penalised confidence intervals, and an absolute difference in days for length of stay; an odds ratio above 1 indicates a higher rate in the Intuity group. ‡ adjusted for age, sex, EuroSCORE II, NYHA class, ejection fraction, and valve size. OR, odds ratio. Comparisons are based on *n* = 192, except paravalvular leak and length of stay (*n* = 190). The adjusted odds ratio for paravalvular leak model has been re-fitted on the corrected count using the full patient-level dataset, giving OR 2.28 (95% CI 0.70–7.72, *p* ‡ = 0.168).

**Table 4:** Paravalvular leak severity.

Severity	Intuity (*n* = 70)	Suture (*n* = 122)
Trace	3 (4.3)	6 (4.9)
Mild	4 (5.7)	2 (1.6)
Moderate or severe	0 (0.0)	0 (0.0)

Severity graded trace/mild/moderate/severe on transoesophageal echocardiography at the end of the procedure; no patient in either group had moderate or severe paravalvular leak. Percentages use the same denominators as Table 3 (69 assessed Intuity, 121 assessed Suture patients; missing in 1 Intuity and 1 Suture patient). This severity breakdown is reported descriptively; the any-grade comparison in Table 3 remains the formally adjusted secondary endpoint.

For additional perioperative complications, re-exploration for chest-wound bleeding occurred in three of 70 Intuity (4.3%) versus two of 122 Suture patients (1.6%; *p* = 0.356), perioperative blood transfusion in 53 (75.7%) versus 84 (68.9%; *p* = 0.326), pneumonia or pulmonary infection in three (4.3%) versus five (4.1%; *p* = 1.000), transient ischaemic attack or stroke in none versus two (1.6%; *p* = 0.534), and pleural haematoma in one (1.4%) versus one (0.8%; *p* = 1.000). Pleural effusion requiring drainage was less frequent in the Intuity group, in one of 70 (1.4%) versus 12 of 122 (9.8%; *p* = 0.034); this was the numerically largest relative difference among the tertiary complications and as it was not adjusted or corrected for multiple comparisons.

### 3.3. Echocardiographic Findings

Postoperative mean transvalvular gradient was lower in the Intuity group, at 8.4 ± 3.1 mmHg versus 13.5 ± 4.7—an adjusted difference of −5.52 mmHg (95% CI −6.80 to −4.25; *p* < 0.001). Peak gradient was likewise lower, at 16.5 ± 5.5 mmHg versus 27.2 ± 9.1, an adjusted difference of −11.50 mmHg (−13.90 to −9.10; *p* < 0.001) (Table 5, Figure 1C,D and Figure 2). Both comparisons were based on 188 of the 192 patients; because the Suture arm was dominated by a single porcine prosthesis (Hancock II) while the Intuity is built on a pericardial platform, and because implanted valve size differed in distribution between groups, this gradient comparison is confounded by prosthesis platform and size and is not isolated to the deployment mechanism alone. Adjusted effect estimates for all endpoints are shown together in Figure 2.

### 3.4. Sensitivity Analysis

Retaining the seven patients who received a 29 mm sutured prosthesis (*n* = 199) did not change any conclusion (Table S2). No endpoint that was significant became non-significant, and none that was non-significant became significant; the adjusted endpoints nearest the threshold, pacemaker implantation, atrial fibrillation, and paravalvular leak, remained non-significant in the primary cohort after re-fitting on the corrected paravalvular leak count (*p* = 0.134, 0.208, and 0.168 respectively); with the additional 29 mm patients, pacemaker and atrial fibrillation likewise remained non-significant (*p* = 0.058 and 0.092), while paravalvular leak could not be re-fitted for this expanded cohort because patient-level data for the seven additional patients were not available, and Table S2 continues to report it descriptively for the all-sizes cohort only. The operative-time, gradient, and length-of-stay estimates were likewise stable.

## 4. Discussion

In 192 patients operated through a right anterior thoracotomy, and after adjustment for baseline differences between groups, the Edwards Intuity valve was associated with shorter aortic cross-clamp and cardiopulmonary bypass times, lower postoperative transvalvular gradients, and shorter hospital stay than conventional sutured bioprostheses, with similar observed in-hospital mortalities (a comparison based on only two deaths that cannot establish equivalence). No secondary safety endpoint reached significance after adjustment, a finding that reflects limited statistical power rather than demonstrated safety: permanent pacemaker implantation and paravalvular leak were both more frequent with the Intuity, and new-onset atrial fibrillation less frequent, the last differing significantly before adjustment but not after.

A question this design could in principle answer, but which we do not resolve here, is whether the valve’s association with the clinical secondary endpoints (length of stay, transvalvular gradients) is mediated entirely by the shorter cross-clamp and bypass times themselves, or reflects a device-specific effect independent of operative time. Cross-clamp and bypass duration were not entered as covariates or mediators in the models for the clinical secondary endpoints, so the present analysis cannot separate a time-mediated pathway from an independent prosthesis effect; this mediation analysis is an important next step for this dataset and is noted explicitly as an unresolved question rather than as an already-answered one.

The cross-clamp reduction we observed is the largest single effect of the valve and matches the mechanism for which it was designed. The adjusted saving of 26.9 min (95% CI 20.1–33.7) closely reproduces the 26 min reduction reported in the prior comparative right anterior thoracotomy series of the Intuity (42 versus 68 min) [21], and sits alongside the Perceval experience through the same access—both a matched comparison [22] and the largest single-centre right anterior thoracotomy series to date, in which sutureless prostheses shortened the median cross-clamp time from 87 to 55 min across 541 isolated replacements [23]—and aligns with the pivotal trials [20,24]. The absolute cross-clamp time achieved here also closely matches a third, single-arm right anterior thoracotomy Intuity series without a conventional comparator (63 ± 11 min) [28]. Within right anterior thoracotomy specifically, the largest registry of rapid-deployment and sutureless valves combined found the same direction of effect, though of much smaller magnitude (54 versus 58 min); the reason is not established, since that comparison pools two valve types and multiple centres rather than isolating the Intuity within a single team [39]. Both cross-clamp and bypass duration independently predict early morbidity after isolated aortic valve replacement [15,16], and cross-clamp time specifically predicts decreased late survival over a twenty-five-year follow-up, whereas bypass time does not [40]. A saving of this magnitude through a constrained access is therefore clinically, not merely statistically, meaningful. This inference nonetheless rests on evidence from other cohorts rather than on any clinical endpoint measured here, since apart from length of stay no clinical outcome differed after adjustment in the present study. A meta-analysis of minimally invasive aortic valve replacement trials found longer cross-clamp and bypass times than sternotomy without any accompanying increase in adverse events, and with hospital stay shorter by almost a day [41], so the harm associated with prolonged times in unselected series may not translate directly to this access. The shorter length of stay observed here is consistent with this mechanism, since bypass duration independently predicted postoperative hospital stay across 1863 isolated aortic valve replacements [15]. This valve-specific benefit is not, however, consistently reproduced elsewhere: the largest matched registry to date found no significant difference in hospital stay between pooled rapid-deployment and sutureless valves and conventional bioprostheses (8.5 versus 8.0 days, *p* = 0.09) [39].

The Intuity was associated with lower postoperative transvalvular gradients, with mean and peak values roughly 40% lower than those of the sutured valves in this cohort; because of the platform and size imbalances described below, this should be read as a gradient advantage observed in this comparison rather than as an established, isolated property of the deployment mechanism. The low gradients themselves are consistent with the single-arm pivotal trial, in which the Intuity’s mean gradient was 10.3 mmHg [20], while the advantage over conventional valves reproduces the direct comparisons—over stented valves in small annuli [42], and in a propensity-weighted comparison of 1688 Intuity against 296 conventional stented valves with median mean gradients of 10 versus 13 mmHg [43]—and reflects the flared subannular frame that widens the outflow tract. Two features of the present cohort qualify this comparison. The Suture arm was dominated by a single porcine prosthesis, the Hancock II (77.9%), whose haemodynamic profile differs from that of pericardial stented valves, and the Intuity arm was concentrated at 25 mm while the Suture arm was concentrated at 23 mm; part of the gradient difference may therefore reflect prosthesis model and implanted size rather than the deployment mechanism alone; a platform-restricted comparison against the pericardial stented valves present in the Suture arm (Perimount/Magna, Avalus, Resilia; *n* = 22), and a valve-size-stratified comparison of gradients, would isolate the deployment-specific component of this effect more directly than the size-adjusted but platform-unrestricted analysis presented here, and both are identified as priority reanalyses of the existing dataset. This gradient advantage is also not universal: the largest multicentre registry, which pools Perceval with Intuity, found essentially no difference in valve gradients against conventional bioprostheses overall [39], though Perceval’s gradients are known to be worse than the Intuity’s specifically [44], which may contribute to a pooled result diluting an Intuity-specific effect. The one opposing signal was a higher unadjusted rate of paravalvular leak with the Intuity, which did not reach significance; reassuringly, every affected patient in both groups had only trace or mild leak on transoesophageal echocardiography, with no moderate or severe leak in either group, so this signal concerns a haemodynamically minor finding rather than the clinically important leak grades most relevant to reintervention risk. This is the recognised trade-off of the deployment mechanism: the CADENCE-MIS randomised trial found significantly more paravalvular leak with the rapid-deployment valve than with full-sternotomy conventional replacement (*p* = 0.027) [45], and a systematic review of 4184 Intuity patients put the rate of severe paravalvular leak requiring reintervention at 3.3% (95% CI 1.7–6.1%) [31]. A broader meta-analysis pooling rapid-deployment and sutureless valves found a higher risk of any paravalvular leak in unadjusted analyses (RR 2.32, 1.53–3.51) that attenuated to non-significance in adjusted estimates (RR 1.25, 0.70–2.23) or for moderate-to-severe leak specifically (RR 2.05, 0.71–5.93) [46]—a pattern closely mirroring our findings.

New-onset atrial fibrillation was less frequent with the Intuity, a difference significant-unadjusted but not after adjustment. The estimate moved towards the null on entry of the NYHA class, the covariate on which the groups were unbalanced, indicating that part of the crude difference reflects baseline composition rather than the prosthesis. Postoperative atrial fibrillation is not benign and is associated with worse perioperative and long-term outcomes [47,48], so a halving of its odds would be of real interest were it confirmed. However, both groups shared the same access, so the sternal-sparing explanation for lower atrial fibrillation after right anterior thoracotomy does not apply here—an access effect otherwise supported by the evidence that minimally invasive valve surgery reduces postoperative atrial fibrillation relative to median sternotomy [49] and that right anterior thoracotomy specifically reduces it even when compared with upper hemisternotomy [50]. The wider evidence is itself inconsistent: one direct Intuity-versus-conventional comparison found more, not less, new-onset atrial fibrillation with the Intuity (34.0% versus 24.1%) [30], while a meta-analysis pooling across valve types found an overall reduction driven entirely by patients undergoing concomitant coronary bypass, which disappeared in isolated replacement—the population studied here [51]. This observation should therefore be regarded as hypothesis-generating only. The imbalance in cardioplegia type ran against rather than towards it, since blood cardioplegia predominated in the Intuity arm and is if anything associated with more postoperative atrial fibrillation [52].

Permanent pacemaker implantation was more frequent with the Intuity, but with only eight implantations the study cannot estimate this risk: the unadjusted rates were 8.6% versus 1.6% (*p* = 0.053), and the penalised adjusted odds ratio is too imprecise to be informative in either direction. The Intuity-arm rate of 8.6% sits toward the lower end of the rates reported for this valve, which span roughly 8–14% across the series [29,30,31], consistent with the subannular frame’s proximity to the conduction system. The wider evidence is more mixed than a single estimate suggests: a large matched analysis of the self-expanding Perceval valve found a tripled pacemaker rate with no accompanying survival benefit [34], a pattern since reproduced under randomisation (11.1% versus 3.6% at one year in 910 patients) [53]. Three independent comparisons using the balloon-expandable Intuity specifically found no significant difference—the largest 6% versus 6% across 1688 Intuity and 296 conventional recipients [38,43,54]—and a meta-analysis pooling across valve types estimated an approximate doubling of risk [51]. The largest matched registry, itself roughly two-thirds Perceval patients, found a closer-to-tripled rate (7.9% versus 2.5%) [39]. This heterogeneity may reflect differences between anchoring mechanisms rather than a uniform rapid-deployment effect.

This study’s strength is its design. Access and surgical team are common to both groups, and the prosthesis is the principal variable distinguishing them from the surgeon’s perspective; however, because prosthesis use also varied with calendar time and cumulative case experience, it is not the only variable that differs systematically between the two groups, and the statement that it is the sole distinguishing variable should be read with that caveat. In contrast, one large matched analysis bundles together [55] the Intuity-versus-sutured comparisons pool surgical accesses [35,37,38] and the multicentre registry pools valve types and accesses [39], and another, restricting both arms to minimal access, still pools hemisternotomy with right anterior thoracotomy [56]. Notably, the large randomised comparison of these valve types excluded right anterior thoracotomy from its protocol, citing variable experience across its forty-seven participating centres [53]. Comparisons of the two principal minimally invasive accesses themselves also remain limited: a recent systematic review and meta-analysis of mini-sternotomy versus right anterior mini-thoracotomy found similar perioperative mortality between accesses but could not analyse prosthesis type in detail because it was inconsistently reported across the included studies [57], underscoring the value of access- and prosthesis-consistent single-team series such as the present one.

### Limitations

Valve choice was not randomised but left to the surgeon, and multivariable adjustment accounts only for confounders that were measured. Second, and most importantly for the primary endpoints, prosthesis use was not randomly distributed in time: Intuity implantation increased in frequency over the study period, and over a three-year period a single team’s operative times fall with accumulating experience of this access, for which a forty-five to fifty case learning period is recognised [13]. Calendar time and case sequence were not entered as covariates in the adjustment model, so part of the observed cross-clamp and bypass-time saving may reflect operator experience or secular improvement rather than a device-specific effect; a case-sequence- or calendar-period-adjusted sensitivity analysis, and stratification of operative times by early versus later study period, are identified as priority reanalyses of the existing dataset that were not part of the original pre-specified plan and are not presented here. Third, the comparator arm was not a single prosthesis but a mixture of six models dominated by the Hancock II, and valve sutures in that arm were fastened routinely with an automated device that itself shortens implantation time; the comparison is therefore against a centre-specific and already accelerated conventional technique, which makes the observed saving conservative but less generalisable. Fourth, follow-up was confined to the index hospital admission, so the late endpoints that matter most for rapid-deployment valves, progression of paravalvular leak, durability, and the survival consequences of pacemaker dependence, cannot be addressed. Fifth, cardioplegia type differed markedly between groups and was not adjusted for, being an intraoperative decision rather than a baseline characteristic; given the size of this imbalance it may also act as a proxy for calendar period, although randomised evidence shows no association between cardioplegia type and mortality, stroke, or atrial fibrillation [52]. Sixth, implanted valve size differed in distribution between groups (concentrated at 25 mm for the Intuity and 23 mm for Suture); size was included as an adjustment covariate in the regression models for gradients, but a size-stratified gradient comparison was not performed and is identified as a priority reanalysis, since transvalvular gradient is a recognised function of labelled valve size independent of prosthesis platform. Seventh, paravalvular leak severity is now reported descriptively (trace versus mild, with no moderate or severe leak in either group; Table 4), which resolves the absence of severity grading in the original submission; the any-grade count for the Suture group was subsequently corrected from 6/121 to 8/121 on the echocardiographic re-review, after the adjusted odds ratio in the previous version of Table 3 had already been estimated from the incorrect count. The full patient-level dataset has since become available and the multivariable Firth model has been re-fitted on the corrected count for the primary (*n* = 192) cohort, giving an adjusted odds ratio of 2.28 (95% CI 0.70–7.72, *p* ‡ = 0.168) in place of the previously reported, pre-correction estimate (2.81, 95% CI 0.81–10.85); the new estimate is consistent in direction and magnitude with the withdrawn one and remains non-significant. Eighth, whether the valve’s association with the clinical secondary endpoints reported here is mediated by the shorter cross-clamp and bypass times themselves, rather than reflecting an independent device effect, was not tested by entering operative time as a covariate or mediator in the secondary-endpoint models; this mediation question remains unresolved. Ninth, the thoracotomy incision length is now reported (skin incision ≤ 6 cm, soft-tissue retractor blade 4–6 cm, with a small rib spreader, under direct vision without video or endoscopic assistance throughout; Section 2.2), which resolves the earlier omission and allows comparison with benchmark series in which incision length is reported as a determinant of exposure; a formal, blinded measurement protocol for incision length was nonetheless not pre-specified, so the figure given here reflects the operating surgeon’s standard practice rather than a systematically recorded per-patient value. The cohort was restricted to annuli within the Intuity’s manufactured size range, so these findings do not extend to patients requiring a prosthesis larger than 27 mm; retaining the seven patients who received a 29 mm sutured valve nonetheless leaves every estimate essentially unchanged (Table S2).

## 5. Conclusions

Within a right anterior thoracotomy, the Edwards Intuity valve was associated with shorter operative times, lower gradients, and shorter hospital stay than conventional sutured bioprostheses, with similar observed in-hospital mortality based on only two deaths, which cannot establish equivalence. No secondary safety endpoint differed significantly after adjustment, but this reflects limited statistical power rather than demonstrated safety: the pacemaker and paravalvular-leak estimates both pointed in the direction the wider literature predicts and were too imprecise to exclude a clinically meaningful difference, and these non-significant comparisons should not be interpreted as evidence that the two prostheses carry comparable risk. Because the primary endpoints are process measures and follow-up did not extend beyond discharge, these results establish operative efficiency rather than clinical superiority. The valve is nonetheless a reasonable option through this access, particularly where reducing operative time is a priority and within the indications for which rapid-deployment valves are already preferred—concomitant procedures, reoperation, or a small aortic root. Broader adoption should await clearer evidence on which patients benefit most, because the large multicentre registries pool valve types and surgical accesses; isolating the contribution of either may still require focused, single-team comparisons with follow-up extending beyond the index admission.

## Figures and Tables

**Table 1:** Baseline characteristics.

Characteristic	Intuity (*n* = 70)	Suture (*n* = 122)	*p*
Age, years	69.06 (5.50)	67.71 (7.22)	0.179
EuroSCORE II	1.00 [0.79, 1.35]	1.17 [0.89, 1.59]	0.032
Ejection fraction, %	58.14 (5.78)	57.84 (7.88)	0.782
Ejection fraction < 50%	10 (14.3)	27 (22.1)	0.185
NYHA class			0.004
I	1 (1.4)	0 (0.0)	
II	46 (65.7)	103 (84.4)	
III	23 (32.9)	19 (15.6)	
Valve size, mm			0.081
19	2 (2.9)	0 (0.0)	
21	11 (15.7)	15 (12.3)	
23	19 (27.1)	53 (43.4)	
25	29 (41.4)	39 (32.0)	
27	9 (12.9)	15 (12.3)	
Male sex	42 (60.0)	79 (64.8)	0.616
Aortic stenosis	69 (98.6)	121 (99.2)	1.000
Crystalloid cardioplegia	26 (37.1)	95 (77.9)	<0.001

Continuous variables are mean ± standard deviation compared with the *t*-test, except EuroSCORE II, given as median [interquartile range] with the Wilcoxon rank-sum test. Categorical variables are *n* (%), compared with the chi-squared or Fisher’s exact test. Percentages for crystalloid cardioplegia use the full group denominators (26/70 and 95/122); cardioplegia type was not recorded in one patient. EuroSCORE II, European System for Cardiac Operative Risk Evaluation II; NYHA, New York Heart Association functional class.

**Table 2:** Operative times.

Outcome	Intuity (*n* = 70)	Suture (*n* = 122)	*p*	Adjusted Difference (95% CI)	*p* †
XCT (min)	61.0 [54.0, 75.0]	91.0 [76.0, 108.5]	<0.001	−26.9 (−33.7 to −20.1)	<0.001
CPB (min)	103.5 [84.2, 115.0]	131.0 [115.0, 153.0]	<0.001	−29.4 (−38.7 to −20.2)	<0.001

XCT, aortic cross-clamp time; CPB, cardiopulmonary bypass time. Per-group values are observed as medians [interquartile range], compared unadjusted with the Wilcoxon rank-sum test. The group effect is the adjusted difference in minutes from multivariable linear regression on log-transformed outcomes with robust standard errors. † Adjusted for age, sex, EuroSCORE II, NYHA class, ejection fraction, and valve size. Adjusted geometric means: 64.0 versus 90.9 min for XCT and 101.9 versus 131.3 min for CPB. Both comparisons are based on *n* = 190; two patients were excluded for missing operative-time data.

**Table 5:** Echocardiographic findings.

Outcome	Intuity (*n* = 70)	Suture (*n* = 122)	*p*	Adjusted Difference (95% CI)	*p* ‡
Mean gradient (mmHg)	8.4 ± 3.1	13.5 ± 4.7	<0.001	−5.52 (−6.80 to −4.25)	<0.001
Peak gradient (mmHg)	16.5 ± 5.5	27.2 ± 9.1	<0.001	−11.50 (−13.90 to −9.10)	<0.001

Values are observed mean ± standard deviation, compared unadjusted with Welch’s *t*-test. The group effect is the adjusted mean difference from multivariable linear regression with robust standard errors; a negative difference favours the Intuity group. ‡ adjusted for age, sex, EuroSCORE II, NYHA class, ejection fraction, and valve size. Adjusted means: 8.2 versus 13.7 mmHg for the mean gradient and 16.0 versus 27.5 mmHg for the peak. Adjustment is for labelled valve size, which is not directly comparable across prosthesis designs; indexed effective orifice area was not available. Both comparisons are based on *n* = 188.

## Data Availability

The data presented in this study are available on reasonable request from the corresponding author. The data are not publicly available owing to institutional privacy restrictions.

## Supplementary Materials

The following supporting information can be downloaded at: https://www.mdpi.com/article/10.3390/life16091573/s1. Table S1: A priori exclusions from the 204 consecutive patients assessed; Table S2: All models re-fitted with the seven patients who received a 29 mm sutured prosthesis retained (*n* = 199). Table S2 is included in full with this revised submission; its earlier absence from the materials provided for review is noted and corrected here.
