# Supplementary material for: Rapid-Deployment Versus Sutured Aortic Valve Replacement Through Right Anterior Thoracotomy: A Single-Team Retrospective Comparison of Operative Efficiency in 192 Consecutive Patients
Source: Life (Basel). 2026 Sep 21;16(9):1573. doi: 10.3390/life16091573 (PMC13608450; doi:10.3390/life16091573)
Supplement: Supplementary file 1 [file life-16-01573-s001.zip › life-4535762-supplementary.pdf]

Supplementary Table S1. Patients excluded before analysis.

PatientID	group	Valve	Valve size	Age	Exclusion reason
S34	Suture	Hancock II 29	29	78	size-29 (no Intuity 29 mm)
S41	Suture	St Jude Regen 23	23	49	mechanical valve
S42	Suture	Hancock II 29	29	72	size-29 (no Intuity 29 mm)
S43	Suture	Hancock II 29	29	53	size-29 (no Intuity 29 mm)
S55	Suture	On-X 23	23	43	mechanical valve
S83	Suture	On-X 23	23	43	mechanical valve
S107	Suture	On-X 27	27	52	mechanical valve
S111	Suture	St Jude Regent 27	27	49	mechanical valve
S122	Suture	Perimount Magna 29	29	60	size-29 (no Intuity 29 mm)
S133	Suture	Hancock II 29	29	75	size-29 (no Intuity 29 mm)
S136	Suture	Hancock II 29	29	74	size-29 (no Intuity 29 mm)
S138	Suture	Hancock II 29	29	61	size-29 (no Intuity 29 mm)

Individual patients excluded from the assessed cohort, with implanted valve and reason for exclusion. Both exclusions were defined a priori (see Methods): five patients received a mechanical rather than a bioprosthetic valve, and seven received a 29 mm sutured prosthesis, a size not manufactured for the Edwards Intuity Elite, so no rapid-deployment comparison was possible.

Supplementary Table S2. Sensitivity analysis: all valve sizes retained.

Outcome	Metric	Primary (n=192)	p	All sizes (n=199)	p
Cross-clamp time (min)	Ratio of geometric means	0.704	<0.001	0.709	<0.001
Cardiopulmonary bypass time (min)	Ratio of geometric means	0.776	<0.001	0.776	<0.001
Mean gradient (mmHg)	Adjusted mean difference	-5.522	<0.001	-5.239	<0.001
Peak gradient (mmHg)	Adjusted mean difference	-11.498	<0.001	-10.943	<0.001
Length of stay (days)	Ratio of geometric means	0.841	0.00879	0.819	0.00243

Outcome	Metric	Primary (n=192)	p	All sizes (n=199)	p
Permanent pacemaker (DDD)	Adjusted OR (Firth)	3.903	0.134	4.143	0.0579
New-onset atrial fibrillation	Adjusted OR (Firth)	0.550	0.208	0.468	0.0921
Paravalvular leak (any)	Adjusted OR (Firth); descriptive only for all-sizes cohort	OR 2.28 (0.70–7.72)	0.168	—	—
In-hospital mortality	Unadjusted, Fisher's exact		1		1

The primary analysis is restricted to patients whose annulus lay within the Intuity's manufactured size range (19–27 mm), i.e. patients for whom either prosthesis was a genuine option. This table repeats every primary model with the seven patients who received a 29 mm sutured prosthesis retained (n = 199), reporting both cohorts side by side. Methods are otherwise identical to Tables 2–4. The operative-time, gradient and length-of-stay estimates are essentially unchanged, and the adjusted endpoints nearest the threshold, pacemaker implantation, atrial fibrillation, and paravalvular leak, remain non-significant in the primary cohort (p = 0.134, 0.208, and 0.168 respectively); pacemaker and atrial fibrillation remain non-significant with the additional patients (p = 0.058 and 0.092). Paravalvular leak (any grade) in the multivariable Firth model has been re-fitted on the corrected count using the full patient-level dataset, giving OR 2.28 (95% CI 0.70–7.72, p = 0.168). All affected patients in both the primary and all-sizes cohorts had trace or mild paravalvular leak only, with no moderate or severe leak.
